# Mr. Williams, on Suspended Animation

**Published:** 1800-03

**Authors:** Allen Williams

**Affiliations:** St. Margaret's Hill, Southwark


### *To the Editors of the Medical and Phyſical Journal.*

GENTLEMEN,

THE following caſe of Suſpended Animation occuring immediately after birth, from the great length of time which elapſed before there were any ſigns of vital action, ſtruck me as curious; and the fortunate iſſue of the attempts to reſtore it, under circumſtances which at firſt appeared hopeleſs, leads me to think that the relation of it may be uſeful.

If you are of opinion that it is ſufficiently intereſting or important to deſerve a place in your valuable publication, you will oblige me by inſerting it.

I am, GENTLEMEN,

Your's, &c.

ALLEN WILLIAMS.

*St. Margaret's Hill, Southwark,*

*January* 18, 1800.

Early

---

Early on Saturday morning, December 14, 1799, I was desired to attend Mrs. J. W. of Borough High Street, Southwark, who, by the account of her husband, was at that time delivered of a child, by the natural efforts of the mother. I arose, and went as expeditiously as possible. Upon my entering the chamber, the lady was in bed, considerably distressed in her mind, from a conviction that this her second child was dead, which was the more unfortunate, as her first child had been still-born. Previously to my arriving at the house, she had been sitting on a night chair, in which I observed a child perpendicularly placed; the head and the placenta immersed in the liquor amnii in the inferior part of the pan, while the legs extended over its superior edge. I immediately removed the child, apparently lifeless, from this situation, in which, from being surrounded by a watery medium, the reception of air into the lungs had been rendered impossible. Upon a careful examination, the actions of the heart and vascular system seemed to have ceased, the face was distended with venous blood, and I feared that the death of an infant could not now be prevented, whose powers were sufficient for her existence previous to birth; and who, only from want of timely professional assistance, had been drowned by that fluid, which before had been her protection. I was led to believe, that the apparent death of the child had been produced by the peculiar circumstances of its birth only, by observing a want of a separation of the cuticle, offensive effluvia, and those other appearances which indicate death, whilst the child is in utero.

The best possible chance which presented itself to my mind for the re-animation of the infant, was the immediate inflation of the lungs; I therefore hastened home, procured a small silver pipe, which, upon my return, I passed into the trachea, distended the lungs with air, and expelled it by a slight compression of the chest and abdomen. I repeated this artificial respiration for five or six minutes, by which time the child had been born above thirty minutes. I then desisted, with a view of ascertaining whether any vital powers were called forth; and the first circumstance which I observed, was an extremely slight tremulous motion, exactly over the left external jugular vein, which I imagine was produced by its contents escaping into the left subclavian, and from thence into the superior cava, from some action of the heart. Almost at that instant, the child took, by her own efforts, a weak inspiration. This first important returning action of life, gave me great hopes that I might prove the happy instrument of resuscitating this infant. I now repeated the distension of the lungs; and a disposition for respiration on the part of the infant being evident, I

thought thought it prudent to commence gentle frictions on the surface of the body with the application of warmth, so as to supply the right side of the heart with blood to continue its actions; I likewise, with my finger moistened in brandy, stimulated the pharynx and glottis, with a view of increasing the frequency of respiration, which by this time took place, once in two or three minutes; and as the child acquired power, it gradually increased.

The infant being naturally of a small size, and of a very delicate constitution, rendered it doubtful for two or three days, whether she could possibly survive the shock which the system had experienced; she, however, then began to take liquid food rather freely, but was not sufficiently strong to suck for ten or twelve days; since which time she takes the breast extremely well, is thriving, and with her mother, in a very healthy state.

The event of this case was certainly highly gratifying to my own feelings, and produced those affectionate sensations in the parents, which are more easily conceived than described.

